# Age‐Dependent Association between Heart Failure and Stroke Risk in Atrial Fibrillation: A Nationwide Cohort Study

**DOI:** 10.1161/JAHA.125.047961

**Published:** 2026-05-06

**Authors:** Eero Jalli, Ville Langén, Jussi Jaakkola, K. E. Juhani Airaksinen, Olli Halminen, Jukka Putaala, Pirjo Mustonen, Jari Haukka, Juha Hartikainen, Miika Linna, Elis Kouki, Mika Lehto, Konsta Teppo

**Affiliations:** ^1^ Department of Internal Medicine, Satasairaala Hospital Pori, Finland and University of Turku Turku Finland; ^2^ Division of Medicine Turku University Hospital and University of Turku Turku Finland; ^3^ Department of Geriatric Medicine Turku University Hospital and University of Turku Turku Finland; ^4^ Cardiac Unit, Department of Internal Medicine Satasairaala Pori Finland; ^5^ Heart Centre Turku University Hospital and University of Turku Turku Finland; ^6^ Aalto University Espoo Finland; ^7^ Department of Neurology Helsinki University Hospital and University of Helsinki Helsinki Finland; ^8^ Turku University Hospital and University of Turku Turku Finland; ^9^ University of Helsinki Helsinki Finland; ^10^ Kuopio University Hospital and University of Eastern Finland Kuopio Finland; ^11^ University of Eastern Finland Kuopio Finland; ^12^ Department of Internal Medicine, Jorvi Hospital HUS Helsinki University Hospital and University of Helsinki Helsinki Finland

**Keywords:** age‐dependency, atrial fibrillation, cohort study, heart failure, ischemic stroke, Atrial Fibrillation

## Abstract

**Background:**

Atrial fibrillation (AF) is a major contributor to ischemic stroke, with risk influenced by age and comorbidities. Age is the strongest risk factor and appears to also modify others, such as vascular disease and female sex. However, data on its impact on additional key risk factors remain limited. We conducted a nationwide retrospective cohort study to examine whether the association between heart failure (HF) and ischemic stroke in patients with AF is age dependent.

**Methods:**

The FinACAF (Finnish Anticoagulation in Atrial Fibrillation) study includes all patients with AF in Finland from 2007 to 2018. Data were collected from healthcare registries encompassing all levels of care. Incidence rate ratios for stroke were calculated comparing patients with HF to those without HF across the entire age spectrum.

**Results:**

We identified 229 565 patients with new‐onset AF. The prevalence of HF was higher among older patients compared with younger patients (38.7% versus 4.3%, respectively). The association between HF and ischemic stroke was highest among younger patients (incidence rate ratio, ≥2.0 for those aged <60 years) and gradually diminished with advancing age, approaching an incidence rate ratio of ≈1.0 in the oldest group (*P*<0.001, for interaction between age and incidence rate ratio). The adjusted absolute rate difference between patients with and without HF remained stable, at ≈1 event per 100 patient‐years, across all age categories.

**Conclusions:**

HF was more strongly associated with ischemic stroke in younger patients than in older individuals with AF, highlighting the importance of considering HF in the decision making about oral anticoagulation, particularly in younger patients.

## Graphical abstract

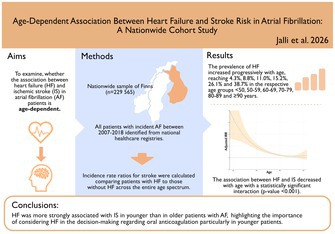

**Nonstandard Abbreviations and Acronyms**
FinACAF: Finnish AntiCoagulation in Atrial Fibrillation
IS: ischemic stroke
OAC: oral anticoagulant

**Clinical Perspective**

**What Is New?**

- This nationwide retrospective cohort study reveals that the stroke risk associated with heart failure is more pronounced among younger patients with atrial fibrillation.

**What Are the Clinical Implications?**

- This finding highlights the need for targeted stroke prevention strategies, as well as proactive screening and management of heart failure in younger patients with atrial fibrillation.

Atrial fibrillation (AF) is the most common sustained cardiac arrhythmia, affecting up to 5.2% of the adult population.^1^ Its prevalence increases with age, reaching over 20% in individuals aged ≥75 years.^1^, ^2^ AF is a major cause of ischemic stroke (IS), but the risk of IS varies significantly between individuals, depending on factors such as age and comorbidities.^3^, ^4^ Accurate stroke risk stratification is crucial for managing patients with AF and determining who would benefit from oral anticoagulant (OAC) therapy.

Age is widely recognized as the strongest risk factor for IS in patients with AF and plays a central role in clinical risk stratification tools.^5^, ^6^ The commonly used CHA_2_DS_2_‐VA score, for instance, assigns 1 point for individuals aged 65 to 74 years and 2 points for those aged ≥75 years, often resulting in recommendations for initiating OAC therapy in patients aged ≥65 years.^7^ In addition to age itself, the prevalence of comorbidities and other stroke risk factors, such as heart failure (HF), increases with age, further elevating stroke risk in older populations.^5^, ^8^ Despite these age‐related differences, the same risk scores are applied uniformly across all age groups, under the assumption that stroke risk factors have consistent relative impact regardless of age. In fact, previous studies have suggested that the components of the CHA_2_DS_2_‐VASc score retain their association with IS across the full age spectrum.^5^

However, recent data indicate that some traditional stroke risk factors in the general population, regardless of the presence of AF, are modified by patient age, indicating that the impact of these risk factors is age dependent.^9^ In patients with AF, a similar phenomenon has been observed concerning the influence of vascular diseases and female sex on IS risk.^10^, ^11^ Nevertheless, there is a paucity of data on whether similar age‐related differences exist with other important stroke risk factors in patients with AF. Given that the overall risk of IS is lower in younger individuals compared with older ones, it is important to understand not only the relative risks associated with these factors but also the absolute risk differences across age groups.

HF is a well‐known risk factor for IS in patients with AF, and it is assigned 1 point in the CHA_2_DS_2_‐VA score. The prevalence of HF has increased, and the trend is also observed among younger patients with AF.^12^, ^13^ Although HF is considered a statistically significant risk factor for stroke across all age groups and is weighted equally in risk stratification and stroke prevention strategies for patients with AF, it remains unclear whether younger individuals with HF have a comparable risk of IS with that of older patients with HF.

Therefore, we conducted a retrospective cohort study using nationwide registry data to investigate whether age‐related differences exist in the association of HF with the risk of stroke in patients with AF.

## Methods

### Data Availability

Because of the sensitive nature of the data collected for this study, requests to access the data set from qualified researchers trained in human subject confidentiality protocols may be sent to the Finnish national register holders (KELA, Finnish Institute for Health and Welfare, Population Register Center, and Tax Register) through Findata (https://findata.fi/en/).

In the interest of research transparency and reproducibility, the analysis code used in this study has been made publicly available on GitHub and permanently archived on Zenodo under DOI: http://doi.org/10.5281/zenodo.17404386. It can be accessed directly online at http://doi.org/10.5281/zenodo.1740438.

### Study Population

The FinACAF (Finnish Anticoagulation in Atrial Fibrillation) study (ClinicalTrials Identifier: NCT04645537; ENCePP Identifier: EUPAS29845) is a nationwide, retrospective cohort study encompassing patients diagnosed with AF across all levels of health care in Finland between 2004 and 2018. Patients were identified through comprehensive national health registries, including hospital inpatient and outpatient specialist records (HILMO), primary care data (AvoHILMO), and the National Reimbursement Register maintained by the Social Insurance Institution of Finland (KELA).

Eligibility for the cohort was based on a recorded diagnosis of AF or atrial flutter, collectively categorized under the *International Classification of Diseases*, *Tenth Revision* (*ICD‐10*) code I48, within the 2004 through 2018 time frame. Individuals were excluded if they had permanently emigrated before December 31, 2018, or were aged <20 years at the time of AF diagnosis.

This subanalysis focuses on a previously defined subset of the FinACAF cohort, consisting of patients with newly diagnosed (incident) AF between 2007 and 2018. To ensure the inclusion of only incident cases, a washout period was implemented by excluding patients with any AF diagnoses recorded during 2004 through 2006, as a medical history shorter than 2 years was considered insufficient to rule out preexisting AF. Furthermore, patients who had filled a prescription for an OAC during the washout period or within 1 year before their initial AF diagnosis were also excluded, to ensure accurate identification of new OAC initiation (Figure S1).

Follow‐up was conducted using two complementary approaches, both commencing from the date of the first AF diagnosis. In the first approach, the entire cohort was followed until the earliest of the following events: first IS; death; or December 31, 2018. This analysis accounted for OAC usage. In the second approach, designed to better reflect stroke risk in the absence of anticoagulation, only periods without OAC treatment were considered. In this case, follow‐up ended at the earliest occurrence of first OAC purchase, IS, death, or the end of the study period. This method has been previously recommended for estimating event rates in untreated populations.^14^, ^15^

Data on baseline comorbidities were drawn from all relevant healthcare registers across care settings.

### Definition of Heart Failure

Patients were identified as having HF if they had relevant diagnostic codes recorded before or at the time of AF diagnosis in hospital or primary care registries (*ICD‐10* codes I50, I11.0, I13.0, or I13.2, or the *International Classification of Primary Care*, *Second Edition* [*ICPC‐2*] code K77), or if they had received HF‐related reimbursement codes in the National Reimbursement Register. Relying solely on hospital‐based data has been shown in previous studies to underestimate the true prevalence of HF. To address this, we used data from multiple national health registries to enhance the accuracy of HF case identification. As the administrative data lacked information on left ventricular ejection fraction, it was not possible to distinguish between HF phenotypes on the basis of ejection fraction. Although the available data did not include detailed information on the pathogenesis or subtype of HF, the definition applied in this study aligns with how HF is defined and used in stroke risk stratification scores and in clinical practice for guiding OAC therapy.

### Outcomes

For patients without a prior IS before or on the date of their initial AF diagnosis, an IS event was defined as the first occurrence of an *ICD‐10* diagnosis code I63 or I64 in the hospital care register following cohort entry. For those with a history of IS before or at the time of AF diagnosis, a new IS event was defined as the date of the first subsequent hospitalization with I63 or I64 listed as the primary diagnosis, provided there was a minimum 90‐day interval from the previous stroke event. Only IS diagnoses recorded in hospital registers were included to ensure the captured events were clinically significant and represented major strokes.

### Study Ethics

The study protocol was approved by the Ethics Committee of the Faculty of Medicine at the University of Helsinki, Helsinki, Finland (no. 15/2017), and research authorization was granted by Helsinki University Hospital (HUS/46/2018). Appropriate approvals were also obtained from the relevant Finnish data authorities: the Social Insurance Institution of Finland (KELA 138/522/2018), the Finnish Institute for Health and Welfare (THL 2101/5.05.00/2018), the Population Register Center (VRK/1291/2019–3), Statistics Finland (TK‐53‐1713‐18/u1281), and the Tax Administration (VH/874/07.01.03/2019). Personal identity numbers were pseudonymized, and the research team received individual‐level data that could not be traced back to specific individuals. Informed consent was not required due to the retrospective, registry‐based design of the study. The study was conducted in accordance with the principles of the Declaration of Helsinki, as updated in 2013.

### Statistical Analysis

We estimated incidence rates and incidence rate ratios (IRRs) of IS using the Poisson regression model with a Lexis‐type data structure based on 2 time scales: follow‐up time from AF diagnosis and age.^16^ This statistical method was chosen to account for patients' age increasing during the relatively long observation period between 2007 and 2018. Age was modeled as a continuous variable using natural splines, with knots placed at the quartiles of the age distribution. We first modeled the crude incidence of IS by age, both over the entire follow‐up period and separately for the follow‐up without anticoagulation. Additionally, separate sensitivity analyses were performed for 1‐year follow‐up. Adjusted IRRs were estimated comparing HF to those without HF by age. We fitted the regression models with interaction terms between age and HF to assess whether the association between HF and the incidence of IS varied significantly with age. In addition to modeling relative differences in stroke rates, we also estimated adjusted absolute rate differences by age associated with HF, compared with the absence of HF. The adjusted analyses included the following variables: sex, age, calendar year, hypertension, diabetes, prior IS or transient ischemic attack, vascular disease, dyslipidemia, prior bleeding, alcohol use disorder, renal failure, liver cirrhosis or failure, cancer, dementia, psychiatric disorders, and income level (divided in tertiles). Additionally, in analyses, also including follow‐up time with OAC use (ie, the first approach described above covering the entire follow‐up), the adjusted analyses also included OAC use, with the follow‐up time split according to OAC exposure. This exposure was considered to start from the first OAC purchase and continue until 120 days after the last drug purchase. The 120‐day interval was chosen since in Finland it is possible to purchase drugs with reimbursement for a maximum of 90 days and an additional 30‐day grace period was allowed to cover possible stockpiling and differences in warfarin dosing. The χ^2^ test, Student's *t* test, and ANOVA were used to compare baseline variables. Statistical analyses were performed with the IBM SPSS Statistics software version 28.0 (SPSS, Inc., Chicago, IL) and R version 4.0.5 (R Core Team, Vienna, Austria; https://www.R‐project.org).

## Results

We identified 229 565 patients with new‐onset AF, 50.0% of whom were women, and their mean age was 72.7 years. The median follow‐up time in the main analysis was 3.24 years. The proportion of women increased with age and older age groups had a relatively higher number of patients in the lowest income tertile compared with younger patients. Patients in the older age groups also had a greater burden of comorbidities and a higher mean CHA_2_DS_2_‐VA score compared with younger patients, although in the oldest patients the prevalence of diabetes and dyslipidemia showed a slight decline (Table). The prevalence of HF was progressively higher in older age groups, reaching 4.3%, 8.8%, 11.0%, 15.2%, 26.1%, and 38.7% in the respective age groups <50, 50–59, 60–69, 70–79, 80–89, and ≥90 years (Table). When comparing younger patients (aged <65 years) to older patients with HF, the proportion of men was higher among the younger group, 75.9% versus 40.1%, respectively. Overall, patients with HF had more comorbidities than those without HF, both among younger and older individuals when analyzed separately (Table S1). The history of myocardial infarction increased steadily with age, ranging from 7.9% to 20.6% among patients with HF, whereas the corresponding proportions among those without HF ranged from 1.4% to 10.8% (Table S2).

**Table 1:** Baseline Characteristics of the Study Cohort According to Age Group

	Age <50 y	Age 50–59 y	Age 60–69 y	Age 70–79 y	Age 80–89 y	Age ≥ 90 y
	n=13 377	n=22 768	n=50 458	n=67 389	n=61 241	n=14 332
Demographics						
Age, y	40.9±7.6	55.8±2.8	65.5±2.8	75.1±2.9	84.6±2.8	93.0±2.5
Female sex	23.7	29.5	38.9	51.3	65.0	76.3
Income tertiles						
First (lowest)	5.2	9.9	19.5	35.4	52.8	64.4
Second	20.0	23.7	33.7	39.3	32.2	23.7
Third (highest)	74.9	66.4	46.8	25.3	14.9	11.9
Comorbidities						
Heart failure	4.3	8.8	11.0	15.2	26.1	38.7
Any vascular disease	5.0	12.8	21.1	30.9	38.0	42.2
Prior myocardial infarction	1.7	4.5	6.8	9.0	11.7	14.6
Diabetes	6.7	15.2	22.2	25.0	23.8	17.9
Dyslipidemia	11.4	31.5	47.6	57.1	54.0	38.0
Hypertension	37.9	60.2	71.6	78.6	82.6	81.9
Prior IS	1.7	4.3	7.9	11.7	14.1	14.6
Abnormal liver function	0.3	0.8	0.9	0.5	0.3	0.1
Chronic kidney disease	1.0	2.2	2.9	3.8	5.5	7.2
Alcohol use disorder	6.9	8.4	6.7	3.1	1.1	0.5
Dementia	0.1	0.2	0.7	3.4	11.0	16.2
Prior bleeding	4.1	6.2	8.9	11.3	13.4	15.2
Risk scores						
Modified HAS‐BLED score	1.3±0.8	1.6±0.9	2.3±1.0	2.8±0.9	2.9±0.9	2.8±0.9
CHA_2_DS_2_‐VA score	0.6±0.8	1.1±1.0	2.0±1.3	3.3±1.4	4.1±1.3	4.3±1.3
CHA_2_DS_2_‐VASc score	0.8±0.9	1.4±1.1	2.4±1.4	3.8±1.5	4.8±1.4	5.0±1.4

Values denote proportions (%) or means±SDs. All differences between age groups *P*<0.001. CHA2DS2‐VASc score indicates congestive heart failure (1 point), hypertension (1 point), age ≥75 years (2 points), diabetes (1 point), history of stroke or TIA (2 points), vascular disease (1 point), age 65‐74 years (1 point), sex category (female) (1 point); CHA2DS2‐VA score same as CHA2DS2‐VASc score but without sex category. Modified HAS‐BLED score indicates hypertension (1 point), abnormal renal or liver function (1 point each), prior stroke (1 point), bleeding history (1 point), age >65 years (1 point), alcohol abuse (1 point), concomitant antiplatelet/NSAIDs (1 point) (no labile INR, max score 8); and IS, ischemic stroke.

OAC initiation was more common in patients without HF than in those with HF (69.8% versus 65.7%, *P*<0.001). When analyzing direct OACs and vitamin K agonists separately, vitamin K agonist use was, in contrast, higher among patients with HF than among those without (Table S3). Ablation therapy as a rhythm control strategy was more common among patients without HF (2.4% versus 0.6%, *P*<0.001; Table S3).

The crude IS rates increased with age, and the increase was observed both in analyses with the entire follow‐up and with the follow‐up without OAC therapy (Figure S2). However, the increase in crude IS rates with age was more pronounced in the analyses without OAC therapy. Patients with HF had consistently higher crude IS rates than patients without HF across the age strata (Figure S2).

**Figure 1:**
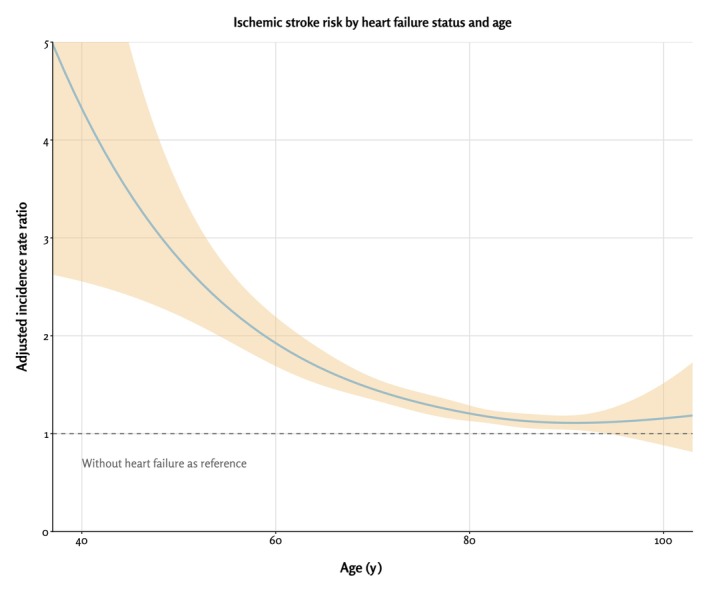
Adjusted IRRs with 95% CIs for ischemic stroke according to age and heart failure status. The broken line represents patient without heart failure as a reference. *P* value for interaction between age and adjusted IRR <0.001. The adjusted analyses included the following variables: sex, age, calendar year, hypertension, diabetes, prior IS or transient ischemic attack, vascular disease, dyslipidemia, prior bleeding, alcohol use disorder, renal failure, liver cirrhosis or failure, cancer, dementia, psychiatric disorders, income level (divided in tertiles) and OAC use. IRR indicates incidence rate ratio; IS, ischemic stroke; and OAC, oral anticoagulant.

The association between HF and IS was highest among younger patients and became lower with increased age, with a statistically significant interaction between age and IRR (*P*<0.001; Figure 1). Patients aged <60 years with HF exhibited an adjusted IRR of ≥2.0, which steadily declined with age, reaching ≈1.0 in the oldest patients. When analyzing only periods without OAC therapy or prior stroke, the results remained consistent, and the decline in IRR was also statistically significant (*P*<0.001; Figures S3 and S4). Results were also consistent in the analysis restricted to 1‐year follow‐up (*P*=0.002; Figure S5).

**Figure 2:**
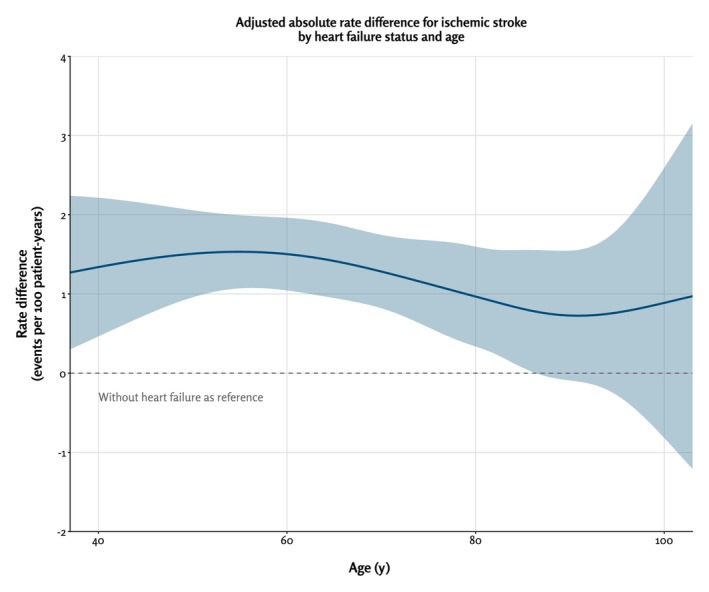
Adjusted absolute rate differences with 95% CIs for ischemic stroke according to age and heart failure status. The broken line represents patients without heart failure as a reference. The adjusted analyses included the following variables: sex, age, calendar year, hypertension, diabetes, prior IS or transient ischemic attack, vascular disease, dyslipidemia, prior bleeding, alcohol use disorder, renal failure, liver cirrhosis or failure, cancer, dementia, psychiatric disorders, income level (divided in tertiles), and OAC use. IS indicates ischemic stroke; and OAC, oral anticoagulant.

The adjusted absolute rate difference associated with HF remained stable, at ≈1 event per 100 patient‐years, across all age categories (Figure 2). These findings were consistent when analyzing only 1 year of follow‐up, periods without OAC therapy, or patients without prior stroke (Figures S6–S8).

## Discussion

This nationwide retrospective cohort study investigated the age‐dependency of the association of HF with IS in patients with AF. Our primary finding is that, in relative terms, the association between HF and IS is substantially stronger in younger patients with AF than in older patients. However, the absolute increase in stroke risk associated with HF was approximately 1 additional event per 100 patient‐years across all age groups compared with patients without HF.

The association between HF and IS was lower with higher age compared with younger individuals, and the difference was statistically significant. This finding is consistent with previous research focused on younger patients with AF or specifically on vascular disease and IS risk in AF.^5^, ^10^, ^17^ It also aligns with the study evaluating risk factors for stroke in the general population, regardless of AF status.^9^ Moreover, although the evidence shows that female sex is also an age‐dependent stroke risk modifier in patients with AF, the pattern is reversed: female sex becomes increasingly associated with IS as age advances.^11^ While earlier research on patients with AF primarily addressed younger age groups, our study stands out by examining these associations across the entire adult age spectrum and specifically focusing on HF, which is one of the most important risk factors.

Although the relative risk for IS in patients with AF and HF was higher in younger individuals, the absolute rate difference was more or less stable across the age spectrum. Therefore, while the absolute overall risk for IS in younger patients is low, the importance of presence of HF is highlighted.

We also observed that crude IS rates increased with advancing age, an anticipated finding supported by prior literature, which recognizes age as the single most powerful predictor of stroke in patients with AF.^2^, ^5^, ^18^

The observed age‐related variation in the association between HF and IS among patients with AF cannot be fully explained by our data and warrants further investigation. Prior literature indicates that, in the general population, patients with HF with reduced ejection fraction tend to be younger than those with HF with preserved ejection fraction.^19^, ^20^, ^21^ A relatively higher proportion of HF with reduced ejection fraction among younger patients, or the greater disparity in comorbidity burden between patients with HF and patients without HF in younger versus older age groups, may partially account for this trend.

Our study demonstrates important implications for stroke prevention and decision making on initiating OAC therapy in patients with AF. While traditional stroke risk factors are pertinent across all age groups in patients with AF, the relative impact of each factor can vary by age. As the findings of our study suggest that the relative stroke risk associated with HF is age dependent and higher in younger patients, accurate risk stratification and individualized risk assessment play a critical role in guiding OAC decisions in this age group. Notably, in 1 previous study, younger age at AF diagnosis has been recognized as a statistically significant risk factor for stroke during a lifetime.^22^ Our findings similarly suggest that HF diagnosed at a young age may serve as an important predictor of stroke among patients with AF. Considering also the fact that the CHA_2_DS_2_‐VA score and current European Society of Cardiology guidelines favor OAC treatment for patients aged >65 years with AF, it is often among younger patients where the presence of individual risk factors, such as HF, makes the difference in initiating OAC treatment.^7^ According to our study, HF appears to be associated with 1 IS event per 100 patient‐years across the entire age spectrum, which exceeds the threshold for initiating OAC therapy defined by guidelines and some recent studies.^23^, ^24^ These findings support the initiation of OAC therapy in all patients with AF with coexisting HF.

As the incidence of AF, HF, and related comorbidities has risen in the general population as well as in younger patients, these findings emphasize the importance of effectively screening for and treating risk factors, especially in younger patients, after an AF diagnosis.^25^ Patients with AF often present with cardiometabolic comorbidities; moreover, evidence indicates a reciprocal relationship in which AF contributes to HF development and, conversely, HF predisposes patients to AF.^26^, ^27^ The results of our study support the conclusion that stroke prevention and the need for OAC therapy should be assessed immediately following an AF diagnosis, including screening for HF and other risk factors, with continued reevaluation during follow‐up if OAC therapy was not initiated initially, given that stroke risk evolves naturally with age and the accumulation of comorbidities. This approach applies both to stroke prevention and to the active treatment of comorbidities, which aligns closely with the “C” component of the AF‐CARE pathway in the current clinical practice guidelines.^7^

The retrospective design based on registry data presents certain limitations that warrant consideration. First, our findings might be influenced by information bias arising from inaccuracies in registry entries, including incomplete or incorrect documentation of diagnostic codes for HF. Importantly, the lack of information on ejection fraction limited the characterization of HF cases. However, the HF definition we applied aligns with the “C” component of the CHA_2_DS_2_‐VA score, enabling us to examine age‐related differences in the relevance of this risk factor. To enhance reliability, we integrated data from registries spanning various levels of health care. Second, the method used to assemble the study cohort may lead to selection bias. Third, because the study is retrospective, the observed associations may not imply causation. Finally, although we adjusted for numerous variables, some residual confounding may persist in the results, particularly given the substantial differences in comorbidities between the youngest and oldest patients. Differences in the use of ablation therapy for rhythm control and in the preference for direct oral anticoagulants over vitamin K agonists may bias the results in favor of patients without HF. Nevertheless, the significant strength of our study is the nationwide coverage through all national health care registries, encompassing uniquely all patients with incident AF in Finland from all levels of care.^28^ The use of the well‐validated hospital care register enhances the reliability of the observed IS outcomes, and the medication information is derived from complete nationwide pharmacy data on redeemed prescriptions.^29^

In conclusion, this nationwide retrospective registry‐based study revealed that HF was more strongly associated with IS in younger patients than in older individuals with AF. These findings underscore the critical need for targeted stroke prevention strategies, as well as proactive screening and management of HF in younger patients with AF.

## Sources of Funding

This work was supported by the Aarne Koskelo Foundation, The Finnish Foundation for Cardiovascular Research, and Helsinki and Uusimaa Hospital District research fund (TYH2019309).

## Disclosures

E.J.: research grant: Turku University Foundation; travel grant: Finnish Cardiac Society. K.T.: research grants: the Finnish Foundation for Cardiovascular Research, Aarne and Aili Turunen Foundation, the Finnish Medical Foundation, the Finnish Foundation for Alcohol Studies, and the Finnish State Research Funding. J.J: none. V.L.: research grants: the State Research Funding of the wellbeing services county of Southwest Finland; speaker: Boehringer‐Ingelheim. O.H.: none. J.P.: speaker: Bayer, Boehringer‐Ingelheim, BMS‐Pfizer, and Abbott; advisory board: Portola, Novo Nordisk, and Herantis Pharma; visiting editor: Terve Media; stock ownership: Vital Signum. P.M.: consultant: Roche, BMS–Pfizer alliance, Novartis Finland, Boehringer Ingelheim, and MSD Finland. J. Haukka: consultant: Research Janssen R&D; speaker: Bayer Finland. M.L.: speaker: BMS–Pfizer alliance, Bayer, Boehringer‐Ingelheim. J. Hartikainen: research grants: the Finnish Foundation for Cardiovascular Research, EU Horizon 2020, and EU FP7; advisory board member: BMS–‐Pfizer alliance, Novo Nordisk, and Amgen; speaker: Cardiome and Bayer. K.E.J.A.: research grants: the Finnish Foundation for Cardiovascular Research; speaker: Bayer, Pfizer, and Boehringer‐Ingelheim. M.L.: consultant: BMS–Pfizer alliance, Bayer, Boehringer‐Ingelheim, and MSD; speaker: BMS–Pfizer alliance, Bayer, Boehringer Ingelheim, MSD, Terve Media, and Orion Pharma; research grants: Aarne Koskelo Foundation, the Finnish Foundation for Cardiovascular Research, Helsinki and Uusimaa Hospital District research fund, and Boehringer‐Ingelheim. E.K.: none.

## Supporting information

Tables S1–S3
Figures S1–S8

STROBE Statement
